# Difference in implant design affects midflexion rotational laxity in cruciate-retaining total knee arthroplasty: a computer navigation study

**DOI:** 10.1186/s40634-023-00652-6

**Published:** 2023-08-21

**Authors:** Takashi Tsuda, Kazunori Hino, Tatsuhiko Kutsuna, Kunihiko Watamori, Tomofumi Kinoshita, Masaki Takao

**Affiliations:** 1 Department of Bone and Joint Surgery, Ehime University, Graduate School of Medicine, Shitsukawa, Toon City, Ehime 791-0295 Japan; 2 Department of Joint Reconstruction, Ehime University, Graduate School of Medicine, Shitsukawa, Toon City, Ehime 791-0295 Japan

**Keywords:** Total knee arthroplasty, Design of implants, Rotational laxity, Midflexion laxity, Navigation system, Cruciate-retaining

## Abstract

**Purpose:**

This study aimed to compare midflexion rotational laxity between two different design concept models of cruciate-retaining total knee arthroplasty: symmetrical surface design of neutral joint line obliquity and asymmetrical surface design of varus joint line obliquity.

**Methods:**

Sixty-three knees that underwent cruciate-retaining total knee arthroplasty were evaluated. Manual maximum passive rotational stress without acceleration was applied to the knees under navigation monitoring. Pre-operative and post-operative internal and external rotational angles were measured at 30°, 45°, 60°, and 90° knee flexion.

**Results:**

The post-operative internal rotational laxity was significantly increased compared with pre-operative levels at 30°, 45°, 60°, and 90° flexion among all subjects (mean 9.7° vs 11.1°, 10.6° vs 11.6°, 11.2° vs 12.9°, and 13.2° vs 14.9°; *p* = 0.01, 0.04, 0.001, and 0.008, respectively). The post-operative external rotational laxity was significantly decreased compared to pre-operative levels at 30°, 45°, 60°, and 90° flexion among all subjects (mean 10.8° vs 6.8°, 12.5° vs 9.4°, 12.8° vs 10.0°, and 11.3° vs 9.5°; *p* < 0.0001, < 0.0001, < 0.0001, and 0.0008, respectively). The post-operative total rotational laxity significantly decreased, compared with pre-operative levels, at 30° and 45° flexion among all subjects (mean 20.4° vs 17.9°, and 23.1° vs 21.1°; *p* = 0.002 and 0.04, respectively). The post-operative total rotational laxity was significantly smaller in asymmetrically designed total knee arthroplasty than in symmetrically designed total knee arthroplasty at 30°, 45°, and 60° flexion (mean 19.3° vs 15.8°, 22.8° vs 18.7°, and 24.4° vs 20.8°; *p* = 0.03, 0.03, and 0.02, respectively), whereas no significant difference was observed at 90° flexion.

**Conclusion:**

Compared to symmetrical surface design, asymmetrical surface design resulted in lower rotational laxity at the midflexion range in cruciate-retaining total knee arthroplasty.

**Level of evidence:**

III.

## Background

Appropriate stability and soft-tissue balance are crucial factors for successful clinical outcomes in total knee arthroplasty (TKA), because joint instability is considered to lead to a significant risk of the need for revision TKA [23, 40]. Recently, several researchers have insisted on achieving anatomical joint stability and normal kinematics [27, 29, 35, 38, 43]. Kinoshita et al. highlighted the high correlation between rotational soft tissue balance and overall kinematics flexion angle between pre- and post-operative TKA [21]. Hence, rotational laxity is considered as one of the key factors for accomplishing proper joint functional reconstruction in TKA.

Image-free navigation systems are optional tools for achieving appropriate bone resection and placement of components [25, 39]. Furthermore, navigation systems are useful for measuring intraoperative midflexion laxity, joint positioning, and knee kinematics [12–17]. Hino et al. demonstrated that the midflexion rotational laxity increased owing to the implantation of posterior-stabilised TKA (PS-TKA) [13], because the cruciate ligaments distribute towards the stabiliser against rotational stress [2, 4]. Nonetheless, there are few reports on midflexion rotational laxity in cruciate-retaining TKA (CR-TKA) [21, 28].

Recently, several studies have investigated the effects of changes in lower-limb alignment and joint line obliquity on the balance between the joint gap and tibial force [8, 9, 34, 37]. However, the effects on midflexion laxity remain unclear. We hypothesised that rotational laxity is affected by differences in the implant design including joint-line obliquity with the preservation of the posterior cruciate ligament (PCL). Thus, the present study aimed to evaluate changes in the midflexion rotational laxity between two different design concept models of CR-TKA: symmetrical surface design and asymmetrical surface design of joint line obliquity in the coronal plane.

## Methods

In total, 88 patients with medial osteoarthritis who underwent CR-TKA using a navigation system (Precision Knee Navigation software version 4.0; Stryker, Kalamazoo, MI, USA) in our hospital were enrolled in this retrospective study. Those with valgus alignment knees, existing inflammatory arthritis, and incomplete rotational laxity data were excluded to unify the conditions of the patients.

**Fig. 1:**
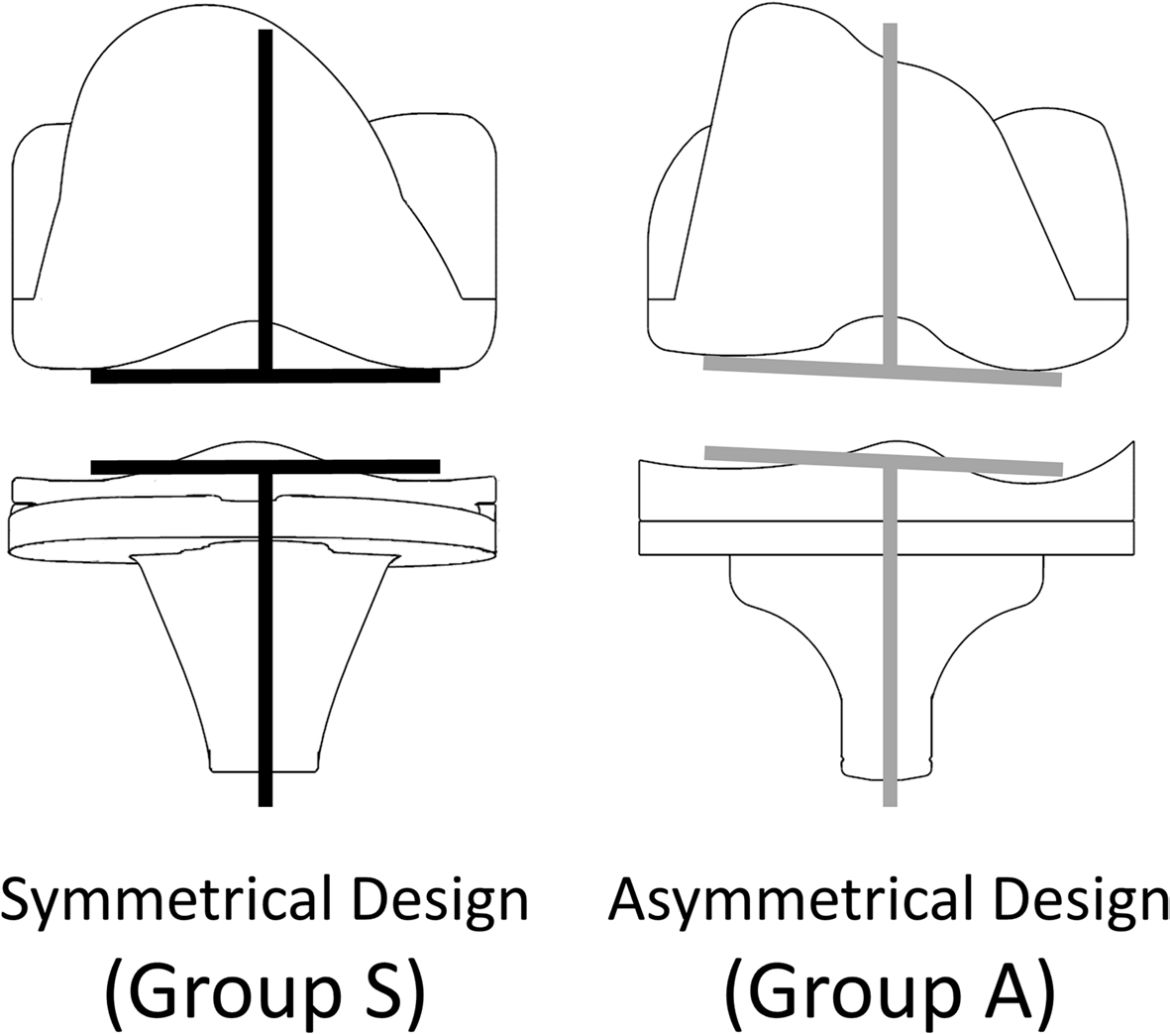
Schematic diagram of two different design concept models of TKA: symmetrical surface design and asymmetrical surface design

Overall, 63 knees of 55 patients with a mean age of 72.4 ± 8.1 years (range: 45–85 years) were enrolled in the study. The average pre-operative hip–knee–ankle angle was 10.6 ± 5.8° in varus knees. The implants used were NexGen CR-Flex, Persona CR (Zimmer, Warsaw, IN, USA, *n* = 37), and FINE Total Knee System (Nakashima Medical, Okayama, Japan, *n* = 26). Further patient characteristics are listed in Table 1. The patients were divided into two groups according to the TKA design concept. NexGen CR-Flex and Persona CR, which induce kinematics to the inherent restraint of implants by adopting the symmetrical surface design of neutral joint-line obliquity between the femoral condyle and the tibial insert, were classified as group S. On the other hand, the anatomical approach that prioritises the kinematics inherent in soft tissues, as the FINE Total Knee System has an asymmetrical design with 3° of joint line obliquity on the insert of the coronal alignment to build in anatomical elements, was classified as group A (Fig. 1) [31, 33]. Both group models adopt multi-radius designs for the femoral component. Meanwhile, there are differences in the radius size between the medial and lateral condyle of group A to reproduce the anatomical joint line. The medial parapatellar approach and measured resection technique were used for all knees. Femoral and tibial joints were resected by mechanical alignment.

**Table 1:** Patient characteristics

	Total (*n* = 63)	Group S (*n* = 37)	Group A (*n* = 26)	*P* value* between groups S and A
Mean age (years) (SD; range)	72.4 (8.1; 45–86)	71.8 (8.0; 45–84)	73.3 (8.3; 61–86)	0.4
Male, n (%)	8 (12.7)	4 (10.8)	4 (15.4)	0.7
Pre-operative hip–knee–ankle angle (°) (SD; range)	10.6 (5.8; 1–27)	10.1 (6.6; 1–27)	11.3 (4.5; 1–20)	0.3
Pre-operative flexion angle (°) (SD; range)	130.9 (12.7; 85–150)	133.0 (8.2; 120–145)	128.3 (16.6; 85–150)	0.5

Values are presented as mean (SD)

*SD* Standard deviation

^*^Mann-Whitney U tests were used for age, pre-operative hip-knee-ankle angle, and pre-operative flexion angle. Fisher's exact test was used for sex

The femoral rotational axis was defined as being perpendicular to the Whiteside line. The tibial rotational axis was set parallel to the line connecting one-third of the tibial tubercle to the centre of the cut surface. Anchoring pins with an infrared signal transducer were fixed into the femur and tibia as reference points, and the joint capsule was temporarily closed with four strands of suture after registration. The investigator gently applied physiologically allowable maximal manual internal and external rotation stress to the knee without angular acceleration, and the mechanical femoral–tibial rotational angle was measured automatically by the navigation system at 30°, 45°, 60°, and 90° of knee flexion. The measurements obtained directly following joint-capsule closure, with the presence of osteophytes, soft tissues, meniscus, and the cruciate ligaments, were defined as the pre-operative record. The articular surfaces of the distal femur and the proximal tibia were resected using the navigation-assisted measured resection technique. The largest sized components without both anteroposterior and mediolateral overhang were selected, after utilising the femoral-sizing guide and tibial-sizing plate. After trial components were placed, the medial–lateral balance of the knee was assessed throughout ROM. When the soft tissue balance was inappropriate, a minimal-released stepped adjustment was applied for accurate ligament balance as necessary: the release of medial collateral ligament, posterior knee capsule or PCL, or bone additional resection.

After confirming that the TKA components and inserts were firmly placed in an appropriate position, the surgical incision was completely closed. Thereafter, the same procedure as described for the pre-operative measurements was repeated to measure and record the rotational angles at 30°, 45°, 60°, and 90° as post-operative rotational laxity. Total rotational laxity was recorded as the sum of the absolute internal and external stress angles. Positive values indicate internal orientation, and negative values indicate external orientation. The accuracy of the navigation system has been established at 0.5°.

The test–retest reliability of these internal and external rotational stress angles indicated that interclass and intraclass correlation coefficients (ICCs) were sufficiently high, with values > 0.9 at 30°, 45°, 60°, and 90°, respectively.

### Statistical analyses

Statistical analyses were performed using JMP software version 16.0.0 (SAS Institute, Tokyo, Japan). The Mann–Whitney U test and Fisher’s exact test were performed to compare groups S and A, whereas the Wilcoxon signed-rank test was performed for pre- versus post-operative comparisons, respectively. In all studies, a probability level of 95% (*p* < 0.05) was considered statistically significant.

Statistical analyses for ICCs were performed using IBM SPSS version 23 (IBM Corp., Armonk, NY, USA). An ICC > 0.81 was considered to be indicative of an almost perfect correlation. The sample size was calculated using Power and Sample Size Calculations software version 3.1.2 (Vanderbilt University, Nashville, TN, USA). After measuring the rotational laxity in the first 10 patients, the mean and standard deviation of the pre- and post-operative TKA data were calculated. To achieve a correlation of δ = 3 and σ = 5 with 80% power and α = 0.05, we determined that a minimum sample size of 39 knees would be required. To compensate for the small sample size, 63 knees were assessed.

## Results

**Fig. 2:**
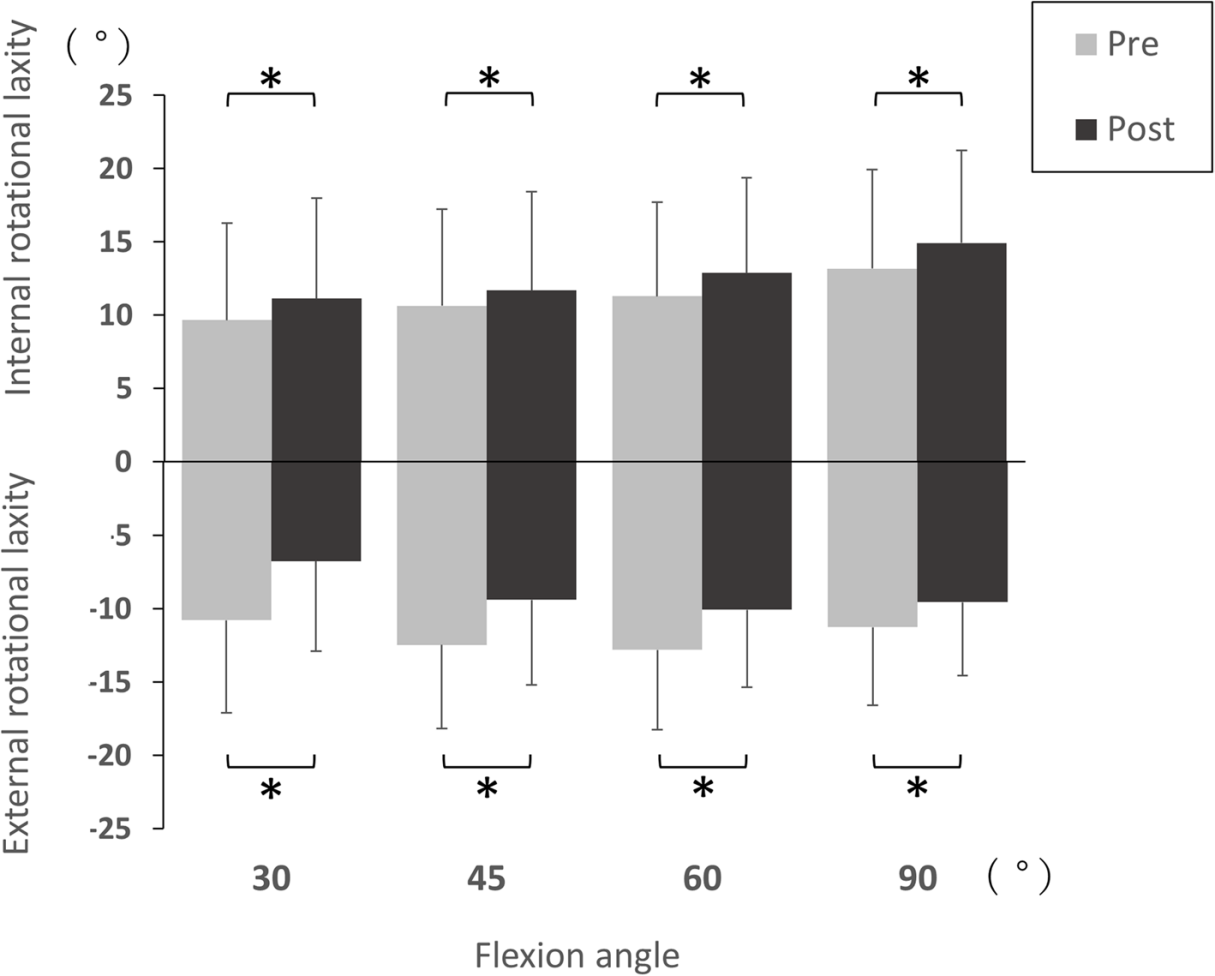
Comparison of internal and external rotational laxity between pre- and post-operative TKA

**Fig. 3:**
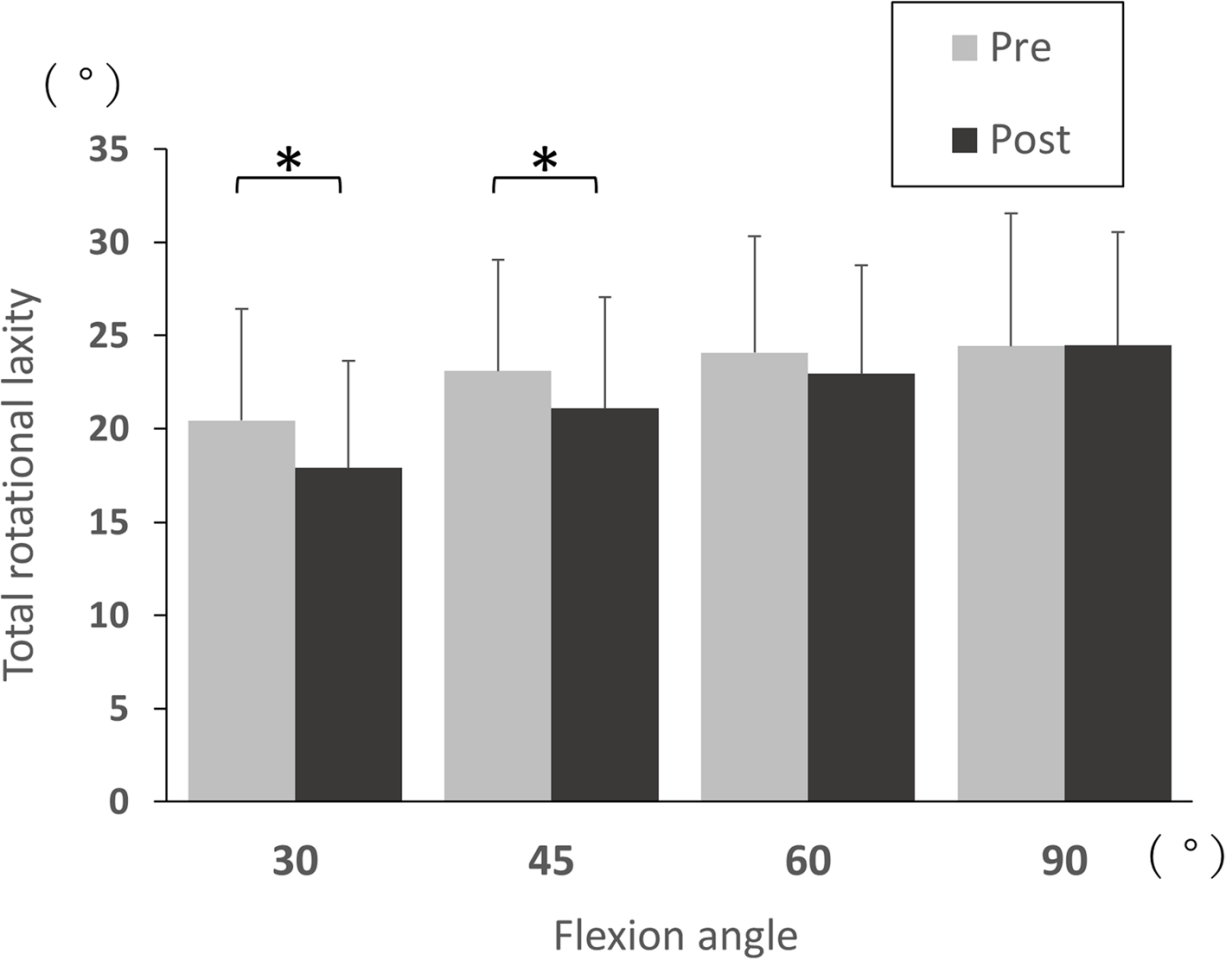
Comparison of total rotational laxity between pre- and post-operative TKA

Table 2 summarises the mean internal, external, and total rotational laxity. The post-operative internal rotational laxity was significantly increased, compared with pre-operative levels, at 30°, 45°, 60°, and 90° of knee flexion (mean 9.7° vs 11.1°, 10.6° vs 11.6°, 11.2° vs 12.9°, and 13.2° vs 14.9°; *p* = 0.01, 0.04, 0.001, and 0.008, respectively; Table 2, Fig. 2). On the other hand, the post-operative external rotational laxity was significantly decreased, compared with pre-operative levels, at 30°, 45°, 60°, and 90° of knee flexion (mean 10.8° vs 6.8°, 12.5° vs 9.4°, 12.8° vs 10.0°, and 11.3° vs 9.5°; *p* < 0.0001, < 0.0001, < 0.0001, and 0.0008, respectively; Table 2, Fig. 2). Moreover, the post-operative total rotational laxity was significantly decreased, compared with pre-operative levels, at 30° and 45° of knee flexion (mean 20.4° vs 17.9°, and 23.1° vs 21.1°; *p* = 0.002 and 0.04, respectively; Table 2, Fig. 3).

**Table 2:** Pre- and post-operative rotational laxity

	Flexion angle (°)	Total			Group S			Group A			*P* value between groups S and A	
		Pre-op	Post-op	*p* value	Pre-op	Post-op	*p* value	Pre-op	Post-op	*p* value		
											Pre-op	Post-op
Internal rotation	30	9.7 (6.7)	11.1 (6.9)	0.01*	10.1 (7.0)	12.0 (6.9)	0.02*	9.1 (6.2)	9.9 (6.9)	0.3	0.8	0.3
	45	10.6 (6.7)	11.6 (6.8)	0.04*	11.2 (7.1)	13.0 (7.1)	0.007*	9.8 (6.0)	9.8 (5.9)	0.9	0.5	0.08
	60	11.2 (6.5)	12.9 (6.5)	0.001*	12.0 (6.7)	14.3 (6.8)	0.0007*	10.2 (6.1)	10.8 (5.7)	0.4	0.5	0.06
	90	13.2 (6.8)	14.9 (6.4)	0.008*	14.3 (6.8)	15.5 (6.8)	0.08	11.6 (6.7)	14.0 (6.8)	0.0002*	0.2	0.3
External rotation	30	10.8 (6.4)	6.8 (6.1)	< 0.0001*	11.1 (5.9)	7.4 (6.3)	< 0.0001*	10.3 (7.1)	5.9 (6.1)	< 0.0001*	0.8	0.4
	45	12.5 (5.7)	9.4 (5.8)	< 0.0001*	12.5 (5.6)	9.7 (5.8)	0.0004*	12.4 (6.0)	8.9 (6.0)	0.0001*	0.9	0.7
	60	12.8 (5.5)	10.0 (5.3)	< 0.0001*	12.9 (5.2)	10.1 (5.2)	0.0002*	12.7 (6.0)	10.0 (5.5)	0.005*	0.9	0.8
	90	11.3 (5.3)	9.5 (5.0)	0.0008*	11.5 (5.2)	9.5 (5.0)	0.002*	10.9 (5.6)	9.7 (5.1)	0.1	0.7	0.8
Total rotation	30	20.4 (6.1)	17.9 (5.8)	0.002*	21.2 (6.2)	19.3 (5.9)	0.1	19.4 (5.8)	15.8 (5.1)	0.003*	0.2	0.03**
	45	23.1 (6.0)	21.1 (6.0)	0.04*	23.7 (6.3)	22.8 (6.1)	0.6	22.2 (5.6)	18.7 (5.1)	0.003*	0.4	0.03**
	60	24.1 (6.3)	22.9 (5.9)	0.4	24.9 (6.3)	24.4 (5.9)	0.9	22.9 (6.2)	20.8 (5.2)	0.2	0.3	0.02**
	90	24.4 (7.2)	24.5 (6.1)	0.6	25.8 (6.9)	25.0 (6.0)	0.8	22.4 (7.2)	23.7 (6.4)	0.2	0.1	0.5

Values are presented as mean (standard deviation)

^*^Wilcoxon signed-rank test

^**^Mann–Whitney U test

**Fig. 4:**
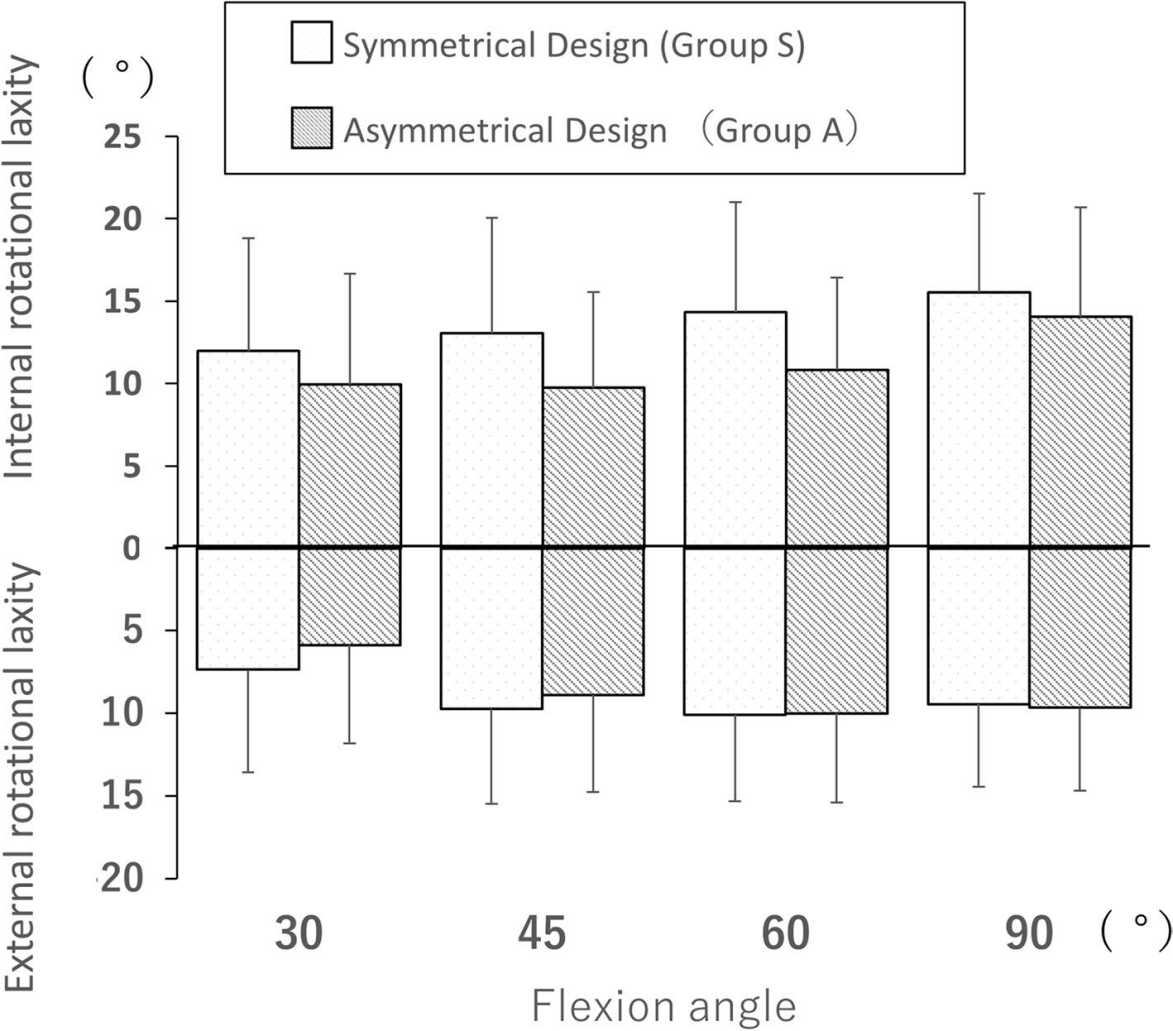
Comparison of post-operative internal and external rotational laxity between groups S and A

**Fig. 5:**
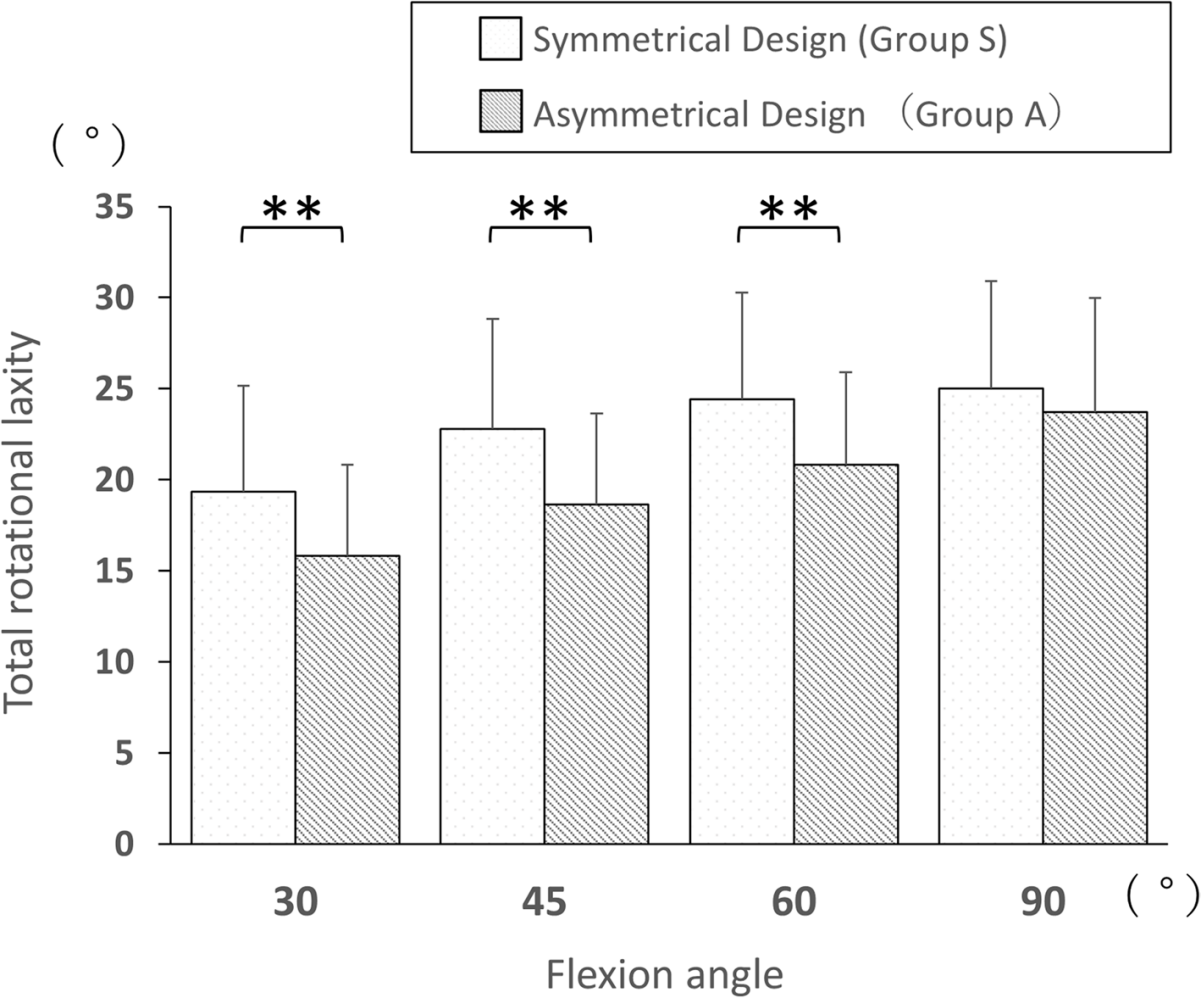
Comparison of post-operative total rotational laxity between groups S and A

Subsequently, the mean difference in post-operative rotational laxity was analysed between groups S and A. No significant difference in internal or external laxity was observed (Table 2, Fig. 4). The total rotational laxity in group A was significantly smaller than that in group S at flexion of 30°, 45°, and 60° (mean 19.3° vs 15.8°, 22.8° vs 18.7°, and 24.4° vs 20.8°; *p* = 0.03, 0.03, and 0.02, respectively; Table 2, Fig. 5). In contrast, no significant difference in total rotational laxity was observed at flexion of 90° among all comparisons.

## Discussion

The most important finding of the present study is that the difference in the design concept affects midflexion rotational laxity in CR-TKA. The results indicated that the rotational laxity at the initial flexion angle of asymmetrically designed implants had significantly decreased, compared with that of the symmetrically designed implants. FINE Total Knee System was developed to reconstruct an anatomical normal knee; the femoral medial condyle has greater radius than the lateral condyle to reproduce 3° of joint line obliquity in coronal alignment. When combined with the insert, it enhances the conformity of the medial condyle joint surface, and the post-operative analysis of kinematics using image-matching has shown tibial internal rotation with medial pivot motion [31, 33]. Furthermore, several cadaveric studies have indicated that the medial structures such as the superficial medial collateral ligament (sMCL), deep medial collateral ligament (dMCL), and posterior oblique ligament resist tibial internal rotational stress during the early stage of flexion [5, 10, 32, 44, 45]. It is inferred from the previous study that the characteristic of FINE seemingly affects the ligament tension pattern, especially with medial structures. As several researchers have emphasised the importance of medial stability for good clinical outcomes in TKA [20, 41, 46], patient-reported outcome measures remain to be investigated further.

The present study also demonstrated that the post-operative rotational laxity at the midflexion range significantly decreased compared with the pre-operative level in CR-TKA. Several studies have shown that the post-operative midflexion gap and varus-valgus laxity in PS-TKA are greater than those in CR-TKA [12, 27], and PCL deficiency resulted in an increased flexion gap [30, 42]. Regarding biomechanics, the PCL is recognised as comprising several small bundles, and the length pattern of each bundle varies throughout flexion [1, 3, 11, 36]. Hence, preservation of the PCL and TKA implantation appear to affect the tension pattern affecting each PCL bundle, resulting in changes in the midflexion rotational laxity.

Contrary to our opinion, Banks et al. [6, 7] reported that CR-TKA had greater rotational laxity than PS or mobile-bearing (MB) TKA, with demonstrated lateral pivot pattern. Their studies were different from our study in that applying the other implant designs, the method of obtaining intraoperative balance varied including soft-tissue releases, and the measurements were performed by image-matching with weight bearing. The clinical significance of the differences between our study and those of Banks et al. leaves room for additional research.

Another key finding of the present study was that no significant difference in total rotational laxity was observed at flexion of 90° among all comparisons. Matsuzaki et al. [28] demonstrated the association between the amount of internal rotation from intermediate to deep flexion and post-operative maximum flexion angle. Kobayashi et al. [22] also reported the correlation of lateral laxity at 80° of flexion with the post-operative flexion angle. These findings indicated that appropriate joint laxity at deep flexion angle was required to obtain favourable maximum flexion angle. Therefore, it seems reasonable to consider that CR-TKA, the focus of our study, ensures the post-operative flexion angle, regardless of whether it is symmetrical or asymmetrical.

The present study has some limitations. First, the operations were performed under general anaesthesia with inflated tourniquet, and navigation data were measured under non-weight bearing conditions. Second, only osteoarthritis knees with varus deformity were covered by the study, and the sample size was limited. Furthermore, the osteoarthritis change varied among the patients, with possible changes in knee kinematics or ligament balance. Finally, despite the quantification and validation of the rotational stress by multiple investigators, measurements were obtained by manual passive stress of the surgeons.

Nevertheless, our study has originality in the assessment of pre- and post-operative midflexion rotational laxity in clinical practice, which has seldom been attempted. Although numerous studies have been conducted to clarify the proper ligament balance in TKA, the relationships between patient outcomes remain controversial [17–19, 24, 26, 46]. Although the present study clarified changes in midflexion rotational laxity in CR-TKA, their association with clinical results has not been fully investigated. Future research could focus on the clinical course and validation of previous results. Our study suggests that variations in joint line obliquity due to the difference in design concepts and the existence of PCL affect midflexion rotational laxity in CR-TKA.

## Conclusions

The asymmetrically designed implant, which reproduces varus joint-line obliquity, resulted in lower rotational laxity in the midflexion range than in the symmetrically designed implant with neutral joint-line obliquity. Furthermore, in CR-TKA, post-operative rotational laxity decreased compared to pre-operative levels in the initial flexion range.

## Data Availability

The datasets used and analysed during the current study are available from the corresponding author on reasonable request.

## Abbreviations

dMCL: Deep medial collateral ligament
ICC: Intraclass correlation coefficient
MB-TKA: Mobile-bearing total knee arthroplasty
PCL: Posterior cruciate ligament
PS-TKA: Posterior-stabilised total knee arthroplasty
sMCL: Superficial medial collateral ligament
TKA: Total knee arthroplasty
